# Understanding Urea Encapsulation in Different Clay Minerals as a Possible System for Ruminant Nutrition

**DOI:** 10.3390/molecules24193525

**Published:** 2019-09-29

**Authors:** Fabrícia C. Silva, Luciano C. B. Lima, Cesar Viseras, Josy A. Osajima, Jarbas M. da Silva Júnior, Ronaldo L. Oliveira, Leilson R. Bezerra, Edson C. Silva-Filho

**Affiliations:** 1 Campus Senador Helvídio Nunes Barros, CSHNB, Federal Unviersity of Piauí, Picos 64600-000, PI, Brazil; briciaquimica@hotmail.com; 2 Interdisciplinary Laboratory for Advanced Materials–LIMAV, Federal Unviersity of Piauí, Teresina 64049-550, PI, Brazil; brandao_lc@hotmail.com (L.C.B.L.); josyosajima@ufpi.edu.br (J.A.O.); 3 Department of Pharmacy and Pharmaceutical Technology, University of Granada, 18071 Granada, Spain; cviseras@ugr.es; 4 Department of Animal Science, Federal University of Bahia, Av. Adhemar de Barros, 500, Ondina, Salvador 40170110, Bahia, Brazil; miguelreges@gmail.com (J.M.d.S.J.); ronaldooliveira@ufba.br (R.L.O.); 5 Department of Animal Science, Federal University of Campina Grande, Avenida Universitária, s/n-Jatobá, Patos 58708110, Paraiba, Brazil; leilson@ufpi.edu.br

**Keywords:** clay minerals, urea, encapsulation

## Abstract

Considering the challenges of urea administration due to the high ureolytic activity of the rumen and the importance of its use, as well as taking into account the relevance of sustainably exploiting the technological potential of biodiversity, this research studies the encapsulation of urea in different clay minerals (palygorskite (Pal), sepiolite (Sep), and Veegum^®^ (V)) as an alternative for use as nonprotein nitrogen (NNP) sources. A method of incorporation was developed in which the encapsulation of urea was proven by X-ray diffraction; fibrous materials, Pal and Sep had similar characteristics due to the decrease in the relative plane intensity (011), suggesting a decrease in the order of their stacking due to the presence of urea on the surface or inside channels. By contrast, V showed a 7.74° reflection shift, suggesting an increase in basal spacing from 11.45 Å in V to 14.88 Å in the sample after urea encapsulation. By thermogravimetry, it was observed that the presence of urea did not change the mass-loss profiles but only increased the percentage of loss in respective events, indicating urea incorporation in the clay minerals. These results provide a promising alternative for administering NNP sources in the ruminant diet.

## 1. Introduction

Urea is a source of nonprotein nitrogen (NPN) that is widely used in the ruminant diet due to its low cost per unit of nutrient and because it is an important alternative that can partially replace the true protein found in a vegetable meal. However, when ingested by an animal, urea is rapidly hydrolyzed, producing ammonia (N–NH_3_) and carbon dioxide. When the ammonia levels absorbed by the rumen exceed the limits in the liver to convert it into urea, ammonia accumulates in the bloodstream, causing intoxication and possibly leading to the death of the animal [1].

Several studies have shown that the ammonia peak in the rumen after feeding depends on the nitrogen sources present in the feed. When urea is supplied, the ammonia peak usually occurs 1 to 2 h after feeding, while for true protein sources, this peak occurs approximately 3 to 5 h after feeding and varies according to the degradability of the substances in the rumen. However, when the rate of degradation of protein in the rumen exceeds the rate of the use of nitrogen compounds in microbial synthesis, the excess ammonia produced in the rumen crosses the rumen wall and could be lost via urine in the form of urea [2].

Researchers have discussed the advantages of formulating diets in which the rate of carbohydrate degradation is synchronized with the rate of protein degradation. In general, the synchronization of protein and carbohydrate degradation in the rumen maximizes the use of degradable protein in the rumen (DPR) and minimizes ammonia loss through the ruminal wall [2].

The high ureolytic activity of the rumen, coupled with the need to adapt animals to feed containing urea, have driven the development of products that allow the slow release of urea into the ruminal environment. One of the obstacles of developing these products is that the alternatives are generally more expensive than urea [1], in addition to other limitations.

Through certain industrial processes, the degradation rate of urea in the rumen can be reduced, and several technologies have been tested in recent decades to control the rate of urea degradation, including starea [3], formaldehyde-treated urea [4], grease protection [5], protection with biuret [6], liquid urea and calcium chloride [7], and polymer-encapsulated urea [8].

Although microencapsulation is considered a relatively new method, it was developed in the 1970s as a packaging technology in the form of thin polymeric coatings for the use on solids, liquid droplets, or gaseous material, forming small particles called microcapsules that were able to release their contents under specific conditions [9] rapidly.

Microencapsulation plays an important role in the chemical, food, agricultural, and pharmaceutical industries [10]. In the food industry, in particular, microencapsulation is widely used for the incorporation of bioactive compounds, such as natural dyes, antimicrobial compounds, antioxidants, and minerals [11]. Microencapsulation also allows the insertion of additives that alter the texture, improve quality, or control certain properties [12]. Desai and Park [13] noted that through its ability to provide finely adjusted controlled release, microencapsulation is not only a method for aggregating substances into a food formulation, but it is a source of wholly new ingredients with unique properties.

The material to be encapsulated is called a filling or core, and the material that forms the capsule is called the encapsulant, covering, or wall capsule [14]. The encapsulant is one of the main factors that influence the stability of the encapsulated compounds [15]. The encapsulants should meet the requirements of having good film-forming properties, low hygroscopicity, low viscosity at high solid concentrations, a mild flavor and odor, easy reconstitution, and low cost, besides being insoluble and nonreactive with the core [16]. 

Products intended for use in the preparation of protectors in the form of excipients or as active ingredients must meet a number of requirements with regard to safety, stability, and high chemical inertia. In the pharmaceutical industry, clay is commonly used because it is chemically and microbiologically innocuous, in addition to its other physical attributes, such as flavor and color. These attributes affect the acceptance by the body, and the texture and water content effect technical processes [17]. Silica and some phyllosilicates, such as talc, kaolinite, smectite, and fibrous clay minerals, are among the most widely used clays in the composition of medicinal minerals [18,19]. These minerals can be used in their natural form or after chemical treatments designed to give them a particular quality. In this way, the special features of palygorskite and sepiolite may make them ideal for use as amino acid molecule complexes with the same success that has been seen for pharmaceutical excipients or active ingredients.

Considering the challenges of urea administration due to the high ureolytic activity of the rumen and the importance of its use and taking into consideration the relevance of sustainably exploiting the technological potential of biodiversity, this research studies the microencapsulation of urea using different clay minerals matrices, aiming the slow release of a NPN source in future for the diet of ruminants.

## 2. Results and Discussion

### 2.1. X-Ray Diffraction (XRD)

**Figure 1:**
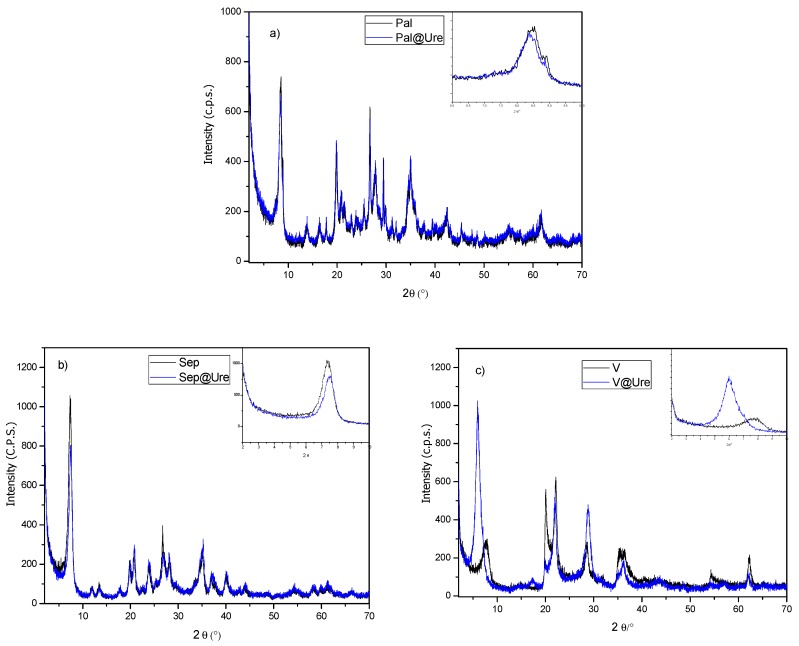
X-ray diffractograms of the clay minerals before and after encapsulation. **a**) Palygorskite (Pal) and Palygorskite with urea (Pal@Ure), **b**) sepiolite (Sep) and sepiolite with urea (Sep@Ure), **c**) Veegum^®^ (V) and Veegum^®^ with urea (V@Ure).

The materials used in this study are clay minerals belonging to two groups: fibrous clays, of which palygorskite (Pal) and sepiolite (Sep) were used, and lamellar clay minerals, of which Veegum^®^ (V) was used. These matrices are multiphase solids in which the fibrous clay minerals consist of the main phases (as indexed from JCPDS 00-021-0958 and 00-029-0863 for palygorskite and sepiolite, respectively) with the presence of small amounts of quartz and illite (as indexed from the JCPDS cards: 01-078-125 and 00-029-1496 for quartz and illite, respectively). The Veegum^®^ was identified as a mixture of phases: montmorillonite (JCPDS 00-002-0037) and cristobalite (JCPDS 01-082-1409). With the encapsulation of the urea molecule in the matrices, it was possible to observe, by X-ray diffraction (Figure 1), different characteristics for each type of clay.

For the fibrous clay minerals Sep (Figure 1a) and Pal (Figure 1b), we observed no significant changes in the characteristic reflections of the matrices used after urea encapsulation, showing that these clay minerals remain unchanged in structure and crystalline organization. These clays remain unchanged due to the nonexpendable characteristics of the Pal and Sep fibrous clay minerals, which do not allow changes in the basal distances. The only characteristic that was observed to change in these materials from the X-ray diffraction technique was a small decrease in the relative intensity of reflection 011 (approximately 8.35° and 7.31° for Pal and Sep, respectively, as seen in the magnifications shown in the graphs of Figure 1a for Sep and Figure 1b for Pal). These observations suggest that the encapsulation of urea in the matrices, superficially, or within the channels of the clays, induces a decrease in their stacking order [20].

In the diffractograms that represent the interaction of V with urea (Figure 1c), was observed a reflection shift related to the basal spacing of approximately 7.74° starting material for the lower angle, i.e., the basal spacing increased from 11.45 Å in Veegum^®^ to 14.88 Å in the sample after urea adsorption encapsulation.

**Figure 2:**
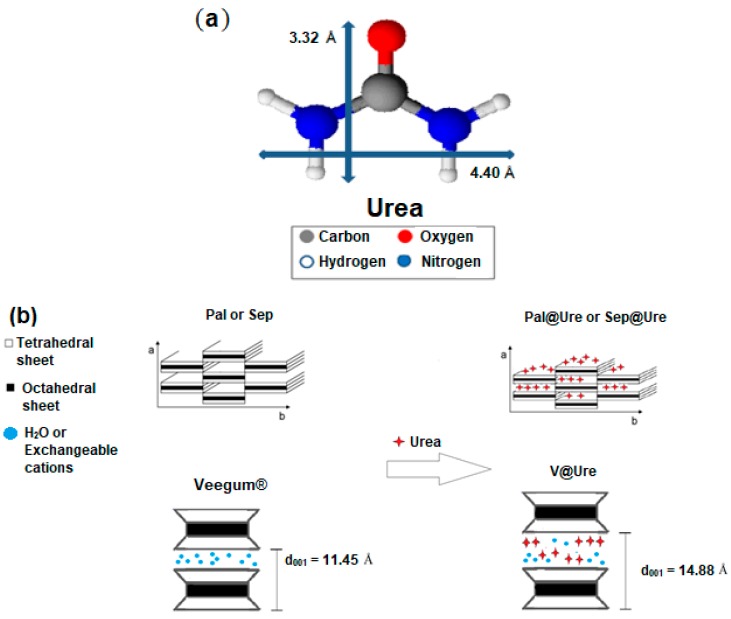
(**a**) Schematic illustration of the molecular structure of urea and its dimensions (calculated using Chem Sketch 12.0 software (ACD/Labs, Toronto, ON, Canada)). (**b**) Scheme for the proposed urea encapsulation.

The increase in basal spacing observed after urea adsorption encapsulation in V is of the same order as the calculated size for the respective organic molecule, as seen in Figure 2a. Therefore, it was possible to construct the proposed urea encapsulation scheme in the studied clays, as shown in Figure 2b, which is supported by subsequent characterizations.

### 2.2. Thermogravimetric Analysis (TG-DTG)

**Figure 3:**
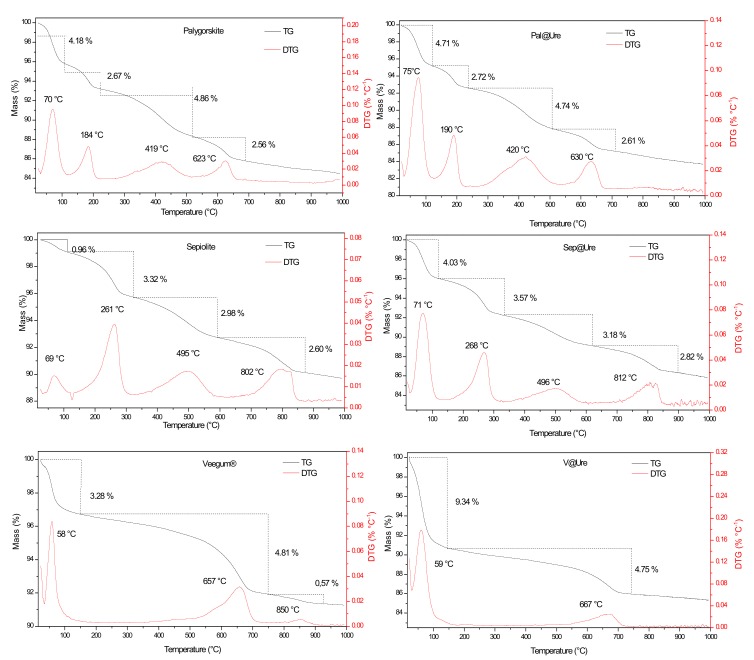
TG and DTG curves of the clay minerals before and after urea encapsulation.

Figure 3 shows the thermogravimetric (TG) and derivatives (DTG) curves of Pal, Sep, and V before and after urea encapsulation. For the two fibrous clay minerals, four mass-loss events are noted that are differentiated by temperature and the respective percentage of mass-loss for each event. The lamellar clay Veegum^®^ undergoes three mass-loss events.

For Pal, the first mass-loss event occurs at approximately 70 °C with an approximately 4.18% mass-loss. This event is associated with the elimination of superficially adsorbed water molecules in the clay structure. The second event, which occurs at 184 °C with a 2.67% mass-loss, is attributed to the loss of zeolitic water molecules from the channels and hydrogen bonds in the fibrous structure. The last two events, at 419 °C (4.86%) and 623 °C (2.56%), are attributed to clay dehydroxylation [21,22].

For Sep, the four mass-loss events have a maximum loss at 70 °C, 261 °C, 495 °C, and 802 °C, with respective mass-loss percentages of 0.96%, 3.32%, 2.98%, and 2.60%. Sep has the same attributes as Pal, and the clay minerals are differentiated by the temperatures and percentages of loss in each event [23,24].

The first mass-loss event observed in the V plot has a maximum at 58 °C and is attributed to the release of surface water molecules, leading to a mass-loss of 3.28%. The loss starts at approximately 510 °C and extends to 900 °C. There are two events, with a DTG peak centered at 657 °C and another with a lower intensity at 850 °C, leading to a total mass-loss of approximately 5.38% due to the elimination of coordinated water that is most strongly bound to octahedral cations and silanol dehydroxylation [25,26].

After the incorporation of organic molecules in the clays (Figure 3), the degradation events in the starting materials continued, but with small changes in the temperatures and percentages of mass-loss due to the incorporating of urea in the materials.

Observing the first mass-loss event of the three clay minerals after encapsulation, we noted a slight shift of the DTG peaks to higher temperatures (these changes are better observed in Table 1). These changes may be associated with two factors. First, the physiosorbed water molecules remaining after encapsulation could form hydrogen bonds with the urea amine groups, which may cause this shift due to the contribution of strengthening intermolecular interactions. Second, the possible release of NH_3_ may contribute to the shift. These events are indicated by the increase in the percentage of mass-loss in the three cases. It is also important to mention that the increase in the percentage of mass-loss related to this event is more pronounced for V, which possibly indicates the higher incorporation capacity of urea due to the expandable characteristic of this clay, as seen in the XRD characterization. This characteristic could lead to the accommodation of a larger amount of urea [27].

A similar effect was observed in the mass-loss events related to the respective condensation and dehydroxylation water losses of each clay, which generally leads to the displacement of the DTG peaks. This displacement may be associated with the small amount of additional energy needed to disrupt the new intermolecular interactions due to urea encapsulation, as well as the additional decomposition of organic matter for CO_2_ release, which contributes to the respective increases in mass-loss percentages. The specific case of Veegum^®^, which does not have a DTG peak at 850 °C, is associated with the coordination of water with the octahedral cations. The last two events may overlap due to the greater interlamellar spacing after the incorporation of urea [28,29,30].

Considering the difference between clay minerals mass-loss before and after urea incorporation, Veegum^®^ was the most efficient, with a total mass difference of 5.46%, followed by sepiolite with a total of 3.74% and palygorskite with a total of 0.52%. This order can be explained by the fact that V is a lamellar clay and intercalation occurs more efficiently in lamellar clays than fibrous clays. Among the fibrous clays, the highest efficiency occurred in sepiolite because the channels in this clay are larger than those in palygorskite.

### 2.3. Scanning Electronic Microscopy (SEM)

**Figure 4:**
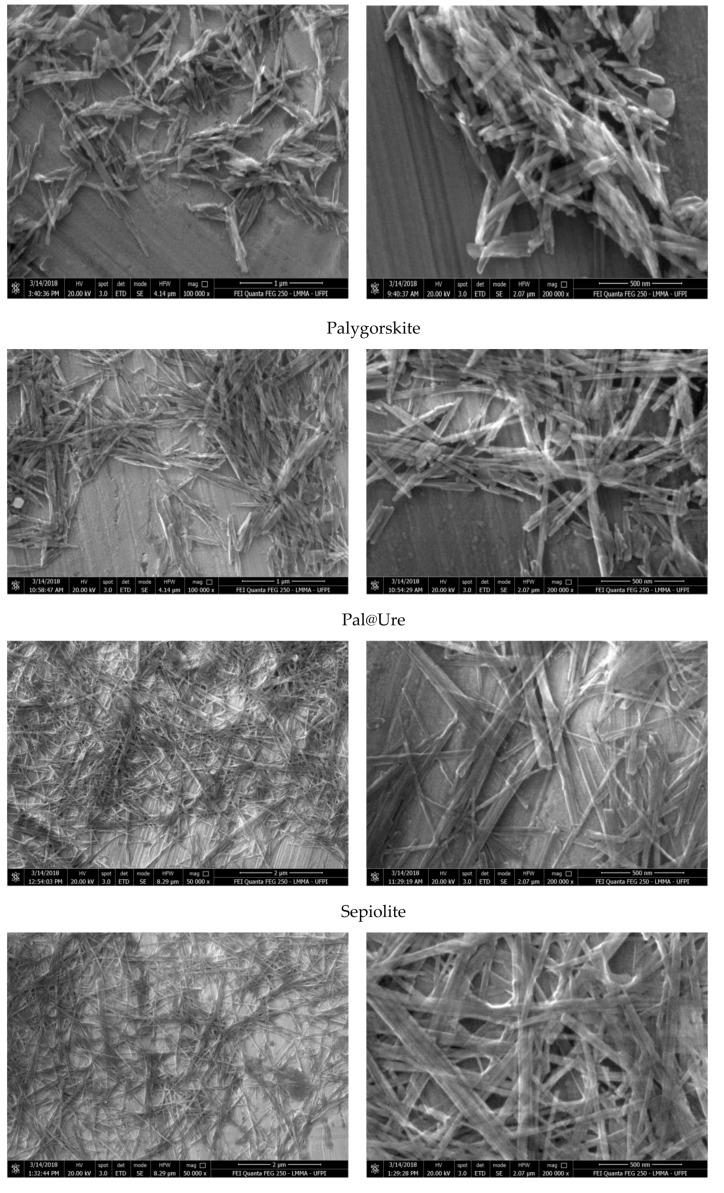

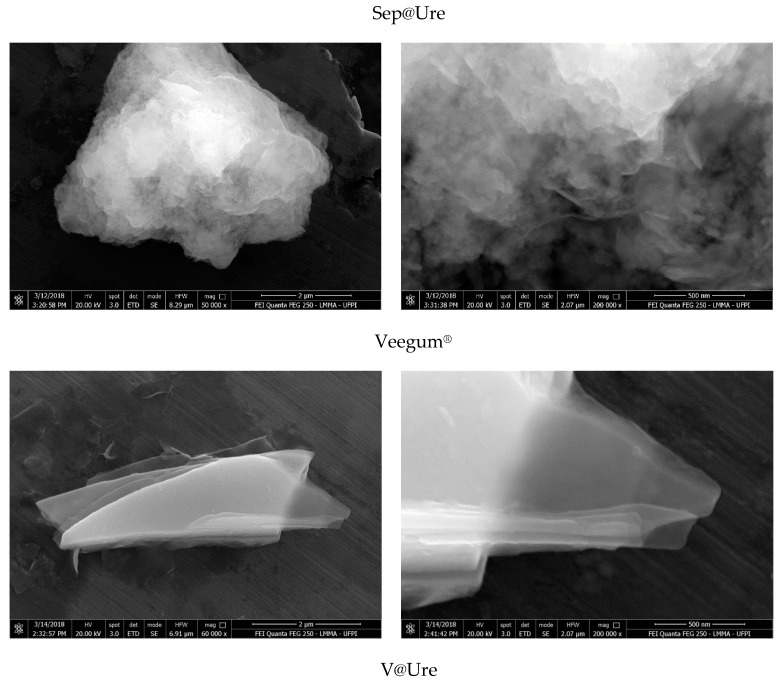
SEM images of the Pal, Sep, and V clay minerals before and after urea encapsulation.

The results obtained by SEM provide information regarding the morphology of the clay minerals before and after urea encapsulation and are presented in Figure 4.

Both Pal and natural Sep have a fibrous structure and form clusters of ribbons and needles [22] or mats and interlaced fibers with varying thicknesses and varying fiber lengths. The histograms show averages of 47 nm and 44 nm for the thickness of Pal and Sep, respectively. After incorporation, more clearly oriented aggregates and average thicknesses greater than of the starting materials were found, specifically at 52 nm and 48 nm for Pal@Ure and Sep@Ure, respectively. These changes are likely to be favored by the presence of the amine groups of urea, which form hydrogen bonds between siloxane (Si–O–Si) or hydroxy (Mg-OH), maintaining the joined fibers [20,31,32].

The V sample had the characteristic lamellar morphology, an aggregation tendency, and the shape of randomly oriented coiled platelets. These platelets became more widely spaced after urea encapsulation, with flat layers that mainly aggregated in one direction. This result corroborated the XRD results, which observed an increase in interlamellar spacing [33].

### 2.4. Fourier Transform Infrared Spectroscopy (FTIR)

**Figure 5:**
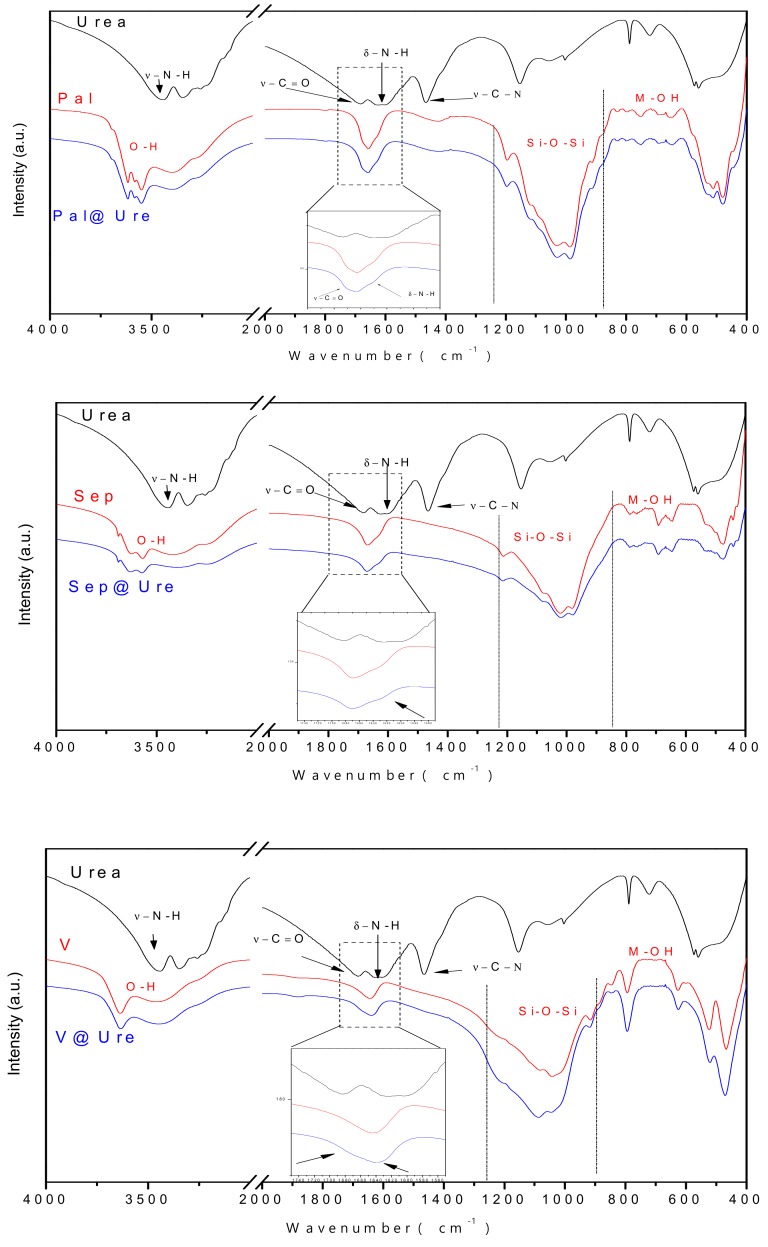
FTIR of urea and the clay minerals before and after urea encapsulation.

The absorption spectra in the infrared region are shown in Figure 5. The three clay minerals exhibited characteristic bands in similar regions and were differentiated by the intensities and bandwidths in the main areas that characterize the materials. These areas are related to tetrahedral sheets Si-O-Si (950–1250 cm^−1^) and M-OH octahedral (M indicates a metal, e.g., Al–Al–OH, Mg–Mg–OH, Al–Fe–OH) that appear in the low-wavelength region as well as bands attributed to hydroxyls and water molecules that appear between 3200–3440 cm^−1^ and 1650 cm^−1^, respectively [22,24,32,34,35,36,37,38,39].

In the FTIR spectrum representing pure urea, the major bands are highlighted, namely, the stretch absorption and NH bond deformation that appear at 3447 cm^−1^ and 1618 cm^−1^, respectively, as well as the stretching frequencies C=O and C–N that appear at 1682 cm^−1^ and 1465 cm^−1^, respectively [40].

The interaction between organic molecules and silicates is generally identified by the observation of changes in the region between 1200 and 2000 cm^−1^. This region coincides with the region where the main bands of the organic molecule appear in this study, and it is observed that although the amount of incorporated urea does not promote the emergence of new bands in the clay spectra, there is a subtle increase of the bands present in the mentioned region. This increase comes from the contributions of the absorptions related to C=O, N–H, and C–N bonds. The band widening between 3200–3440 cm^−1^ is also observed in clay minerals after encapsulation, and this change is associated with the contribution of the N–H stretch vibration, confirming the presence of urea in the final materials [20,32,41,42].

### 2.5. Zeta Potential ζ

The potential zeta values found for the samples in this work are presented in Table 2.

**Figure 6:**
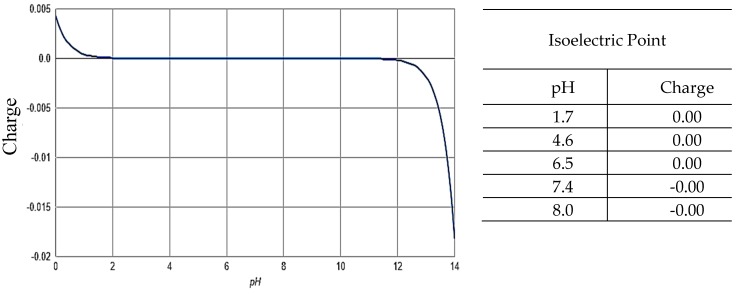
The isoelectric point of urea (calculated using the software MarvinSketch 18.8, ChemAxon, Cambridge, MA, USA).

From the results presented in Table 2, the interaction between the clay minerals and urea has a nonelectrostatic nature since there are small differences in the load surfaces of the materials that make the ζ values more positive after encapsulation. In addition, urea has no ionizable atoms, causing the molecule to remain neutral even in media with different pH values, as seen in Figure 6.

The small change observed in the zeta potential can be attributed to electric double layer compression as a result of nitrogen coordination between urea amine groups with surface silanol (Si–OH) groups through water bridges in the case of fibrous clays and the increase in interlamellar spacing after the incorporation of organic molecules in the case of Veegum^®^ [33,34].

## 3. Materials and Methods

### 3.1. Materials

Purified pharmaceutical degree Veegum^®^ (Veegum HS^®^, VHS) (V) was purchased from Vanderbilt Company (Norwalk, CT, USA). The Sepiolite samples from Vicálvaro (Madrid, Spain) (Sep) were kindly gifted by TOLSA S.A. (Madrid, Spain). Pharmaceutical grade palygorskite (Pharmasorb^®^ Colloidal) (Pal) from Basf SE (Ludwigshafen, Germany) were used as received, and urea (Sigma-Aldrich, São Paulo, SP, Brazil) was used in solutions prepared with deionized water.

### 3.2. Urea Encapsulation into Clay Minerals

To incorporate urea into the clay minerals under study, an adsorption method was used in which 1.0 g of palygorskite (Pal), sepiolite (Sep) or Veegum^®^ (V) was suspended in 30.0 mL of urea (Ure) aqueous solution prepared at a concentration of 0.01 mol L^−1^. The experiment was performed in triplicate, and the suspensions were maintained at a temperature of 37 ± 1 °C for 48 h under constant agitation. Then, solids were separated by filtration, washed to remove excess encapsulated urea, and dried at 50 °C for 24 h. The samples after encapsulation were called Sep@Ure, Pal@Ure, and V@Ure.

### 3.3. Characterizations

The techniques used to characterize the materials before and after adsorption were X-ray diffraction, thermogravimetric analysis, scanning electron microscopy, infrared, and zeta potential. The dimensions of urea molecule were estimated by using a freeware version of Chem Sketch 12.0 software (ACD/Labs, Toronto, ON, Canada).

#### 3.3.1. X-Ray Diffraction (XRD)

X-ray diffraction was performed on a XRD-6000 A (Shimadzu, Nakagyo-ku, Kyoto, Japan) operated at 40 kV and 30 mA, varying 2θ in the range between 2° and 70°. The scanning speed was 2° min^−1^. A CuKα radiation source was used, and the wavelength was 154.06 pm.

#### 3.3.2. Thermogravimetric Analysis (TG-DTG)

Thermogravimetric analyses of the samples with initial weights of 10.0 ± 1.0 mg was obtained on a SDT Q-600 V20.9 Build 20 (TA instrument, New Castle, DE, USA) at a temperature ranging from 25 to 1000 °C with a heating rate of 10 °C min^−1^ and a constant nitrogen flow of 10mL·min^−1^ and using alumina pan.

#### 3.3.3. Scanning Electron Microscopy (SEM)

Scanning electron microscopy was performed using FEI QUANTA 250 Field Emission Gun (FEG) equipment (Thermo Fisher Scientific, Eindhoven, the Netherlands). Samples were dispersed in isopropyl alcohol, sonicated for 8 min, and dripped. Samples were covered with aluminum foil. The drying time was approximately 1 h. Micrographs were obtained at 20 kV and spot size 3.

#### 3.3.4. Infrared Spectroscopy (FTIR)

Fourier transform infrared spectra of the samples were obtained using a PerkinElmer FTIR spectrometer, model 2400 (PerkinElmer, Waltham, MA, USA). The 1% sample KBr pellet method was used. We performed 60 scans in the region from 4000 to 400 cm^−1^ with a 4 cm^−1^ resolution.

#### 3.3.5. Zeta Potential

The surface loading properties of the materials were determined from their zeta potential (ζ) values in aqueous suspension (0.05%, w/v, pH 6.4) on a Malvern Zetasizer Nano instrument (Malvern Panalytical Instruments, Malvern, UK).

## 4. Conclusions

The encapsulation process was satisfactory for obtaining new materials of urea/clay minerals. The characterization of these materials proved the effectiveness of urea encapsulation in the proposed clays (palygorskite, sepiolite, and Veegum^®^). The XRD results indicate two different behaviors of urea encapsulation dependent to the morphology corroborated by SEM (fibrous to Pal or Sep and lamellar to V), specifically, Pal and Sep had similar characteristics that suggest a decrease in the order of their stacking due to the presence of urea on the surface or inside channels, while V showed a shift in d001 peak that suggest an increase in basal spacing from 11.45 Å to 14.88 Å after urea encapsulation. The thermal analyses showed increases in the percentage of loss-mass events, which indicated the urea incorporation with a higher encapsulation rate for Veegum^®^, which could be explained by its expansive characteristic that allows intercalation of organic molecules in the interlamellar space. The FTIR spectra and zeta potential results also support the encapsulations by differences observed in respective results.

Thus, these results provide a promising alternative for planning the administration of NNP sources in the ruminant diet. This line of research, which involves the bioavailability of nitrogen by a nonprotein source, is promising, and its continuity may offer an alternative to improve the diet of cattle, as well as reduce feed costs.

## Figures and Tables

**Table 1:** Temperatures and percentages of mass-loss in each event demonstrated in the thermogravimetric curves.

Clay Minerals		Event 1	Event 2	Event 3	Event 4
Palygorskite	Without Urea	70 °C	184 °C	419 °C	623 °C
		4.18%	2.67%	4.86%	2.56%
	With urea	75 °C	190 °C	420 °C	630 °C
		4.71%	2.72%	4.74%	2.61%
Sepiolite	Without Urea	69 °C	261 °C	495 °C	802 °C
		0.96%	3.32%	2.98%	2.60%
	With Urea	71 °C	268 °C	496 °C	812 °C
		4.03%	3.57%	3.18%	2.82%
Veegum^®^	Without Urea	58 °C	657 °C	850 °C	-
		3.28%	4.81%	0.57%	-
	With Urea	59 °C	667 °C	-	-
		9.37%	4.75%	-	-

**Table 2:** Zeta potential of clays before and after urea encapsulation.

Material	Zeta Potential of Clays (mV)	Zeta Potential of Clays with Urea (mV)
Palygorskite	−14.1	−13.6
Sepiolite	−22.0	−18.7
Veegum^®^	−34.4	−32.7
